# The role of fibrillin and microfibril binding proteins in elastin and elastic fibre assembly

**DOI:** 10.1016/j.matbio.2019.06.006

**Published:** 2019-11

**Authors:** Alan R.F. Godwin, Mukti Singh, Michael P. Lockhart-Cairns, Yasmene F. Alanazi, Stuart A. Cain, Clair Baldock

**Affiliations:** Wellcome Centre for Cell-Matrix Research, Division of Cell-Matrix Biology and Regenerative Medicine, School of Biological Sciences, Faculty of Biology, Medicine and Health, University of Manchester, Manchester Academic Health Science Centre, Manchester M13 9PT, UK

## Abstract

Fibrillin is a large evolutionarily ancient extracellular glycoprotein that assembles to form beaded microfibrils which are essential components of most extracellular matrices. Fibrillin microfibrils have specific biomechanical properties to endow animal tissues with limited elasticity, a fundamental feature of the durable function of large blood vessels, skin and lungs. They also form a template for elastin deposition and provide a platform for microfibril-elastin binding proteins to interact in elastic fibre assembly. In addition to their structural role, fibrillin microfibrils mediate cell signalling via integrin and syndecan receptors, and microfibrils sequester transforming growth factor (TGF)β family growth factors within the matrix to provide a tissue store which is critical for homeostasis and remodelling.

## Introduction

Elastic fibres are essential components of all mammalian elastic tissues such as blood vessels, lung, joints and skin. The main components of elastic fibres are elastin and fibrillin, however an array of other matrix proteins are required for their correct assembly and function [1,2]. Elastic fibre proteins are critically important in the development and homeostasis of elastic tissues both in terms of their key roles in linking cells and matrix macromolecules [3] and in the extracellular regulation of TGFβ family member growth factors [4]. Fibrillin assembles to form beaded microfibrils [5] and the formation of elastic fibres requires a fibrillin microfibril scaffold for the correct deposition of elastin. Fibrillin also interacts with other elastic fibre proteins including the fibulins and latent TGFβ binding proteins to support elastic fibre assembly and function. In this review, we will describe our understanding of the function of fibrillin and fibrillin microfibrils, focusing on its structure, assembly and interaction with other elastic fibre proteins as well as their functional role in elastogenesis.

## Fibrillin microfibrils

### Fibrillin domain structure

**Fig. 1:**
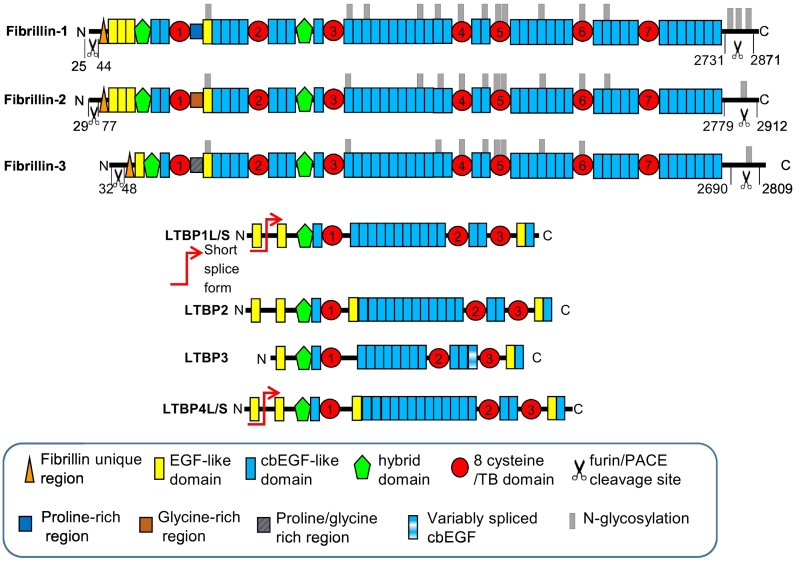
Cartoon representation of the domain structures of the fibrillin superfamily members including fibrillins1–3 and LTBPs1–4.

The fibrillin superfamily family is composed of three fibrillin isoforms, fibrillin1–3, each encoded by a separate gene [[6], [7], [8], [9]], and the related extracellular matrix (ECM) proteins the latent transforming growth factor (TGF)β binding proteins (LTBPs)1–4 (Fig. 1). The domain structure of the fibrillin superfamily consists primarily of arrays of epidermal growth factor-like (EGF) domains interspersed with TGFβ-binding like (TB) domains and hybrid domains [6]. The three fibrillin isoforms are highly homologous to each other with differences including a proline rich region in fibrillin-1 which in fibrillin-2 is glycine rich and in fibrillin-3 is proline and glycine rich. Of the 47 EGF domains in fibrillin, 43 are calcium binding (cb) [6]. There are seven TB domains (also referred to as 8 cysteine domains) which are unique to the fibrillin superfamily. They have a globular structure [10,11] and domain TB4 contains an RGD motif which is involved in binding to α_5_β_1_ α_v_β_3_ and α_v_β_6_ integrins and essential for the interaction between fibrillin-1 and the cell surface [[12], [13], [14], [15]]. Hybrid domains have structural similarity to both EGF and TB domains [6,16,17] and there are two hybrid domains in fibrillin. The fibrillins have unique N- and C-termini which are both proteolytically processed by furin, essential for the assembly of fibrillin into microfibrils [[18], [19], [20]]. The processed C-terminal peptide, also known as asprosin, has been shown to be involved in glucose release from the liver [21]. The fibrillins undergo several other post translation modifications, fibrillin-1 has 14 predicted glycosylation sites and there are 12 sites in fibrillin-2 and 10 sites in fibrillin-3. Fibrillin-1 can also be phosphorylated at serine 2702 by FAM20C [22] but the function of phosphorylation has not yet been investigated.

### Supramolecular organisation into microfibrils

Fibrillin microfibrils are beaded filaments with ~56 nm periodicity mainly composed of fibrillin molecules [23]. The microfibrils are polar polymers which linearly assemble through interaction between the N- and C-termini of adjacent fibrillin molecules [24]. Lateral association also occurs and is driven by homotypic interaction between the termini to form microfibrils [[25], [26], [27]]. Scanning transmission electron microscopy (STEM) mass mapping has shown that microfibrils have a mass of ~2500 kDa per repeat [28] which is consistent with 8 fibrillin molecules in cross section which is supported by 3D reconstructions [29] and 2D images of microfibrils viewed in cross section [30,31]. After linear and lateral assembly, microfibrils are then further stabilised by the formation of transglutaminase cross links between their N- and C-termini [32].

**Fig. 2:**
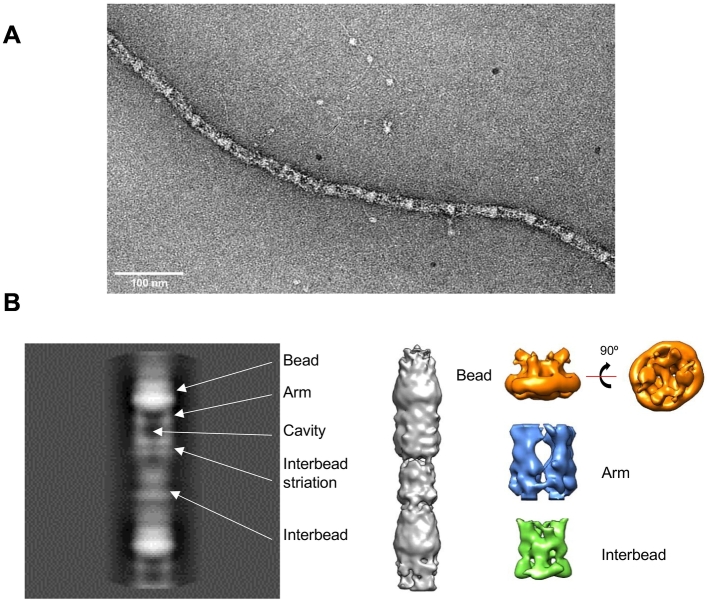
(A) Negative stain transmission electron microscopy (TEM) image of a microfibril extracted from bovine ciliary zonule tissue. Scale bar = 100 nm. (B) The left panel shows an aligned single particle average of a fibrillin microfibril repeat. Box size = 102 × 102 nm. In grey is a 3D reconstruction of a fibrillin microfibril. Separate reconstructions of the bead, arm and interbead regions of the microfibril are shown.

Fibrillin microfibrils are flexible and have a hollow cylindrical appearance when visualised in three dimensions by electron microscopy with single particle image analysis, this is consistent with tube-like structures observed using quick freeze deep etch electron microscopy [29,33]. The microfibril can be described by three distinct regions based on their banding pattern, these have been termed the bead, arms and interbead regions [34,35] (Fig. 2). The microfibril bead region has a dense interwoven core surrounded by a ring structure which forms four arms which combine into the denser interbead region [29]. The bead region contains the N- and C-termini of the fibrillin molecules [24,153]. The microfibril is pseudo-symmetrical, supporting models that describe eight fibrillin molecules in cross-section [29,31]. Extracted microfibrils have a 56 nm repeating periodicity [34], similar to the 50–60 nm periodicity measured in tissues and can reversibly extend to ~80 nm [36,37]. Microfibril diameter is typically ~20 nm and does not vary from tissue to tissue [35] suggesting that this is carefully controlled during assembly, either limited by the packing of fibrillin molecules or by microfibril associated binding proteins.

How fibrillin molecules are organised into mature microfibrils however is still unclear. Extracted and recombinant fibrillin molecules have a length of ~148 nm [5,25] which does not reconcile readily with the periodicity of 56 nm seen for mature microfibrils. Therefore, several models of molecular organisation have been proposed which fall into two categories: the molecular folding model where a single molecule spans one 56 nm repeat [38,154] and staggered models where the fibrillin molecules are staggered by a half or a third and span two or three repeats [11,39]. Both packing models can explain antibody localisation of fibrillin regions within the microfibril, therefore higher resolution imaging or further labelling is required to provide resolution of the microfibril structure.

## Microfibril assembly

### Role of Heparan Sulphate in microfibril assembly

Although the precise mechanism of microfibril assembly is not fully understood, there is considerable evidence supporting the molecular and cellular interactions involved. Microfibril assembly consists of multimerization of the fibrillin molecules, deposition into the ECM, then recruitment of other microfibrillar components such as elastin and MAGP-1. It has been demonstrated that fibrillin monomers can multimerize by interactions between the N- and C-termini, as well as N—N and C—C interactions [25,26,40,41], also the interaction strength is increased between multimers of N- and C-terminal fragments [27]. Fibrillin expression and secretion alone is not sufficient to support microfibril formation, as demonstrated with fibrillin expressing HEK293 cells which have to be co-cultured with fibroblasts for matrix fibrillin microfibril deposition [42]. Other ECM molecules or specific cellular interactions are also required.

A key driver of microfibril assembly is interaction with Heparan Sulphate (HS). Heparan sulphate proteoglycans have a key role in the elastic fibre interactome [43], and HS has a strong interaction with fibrillin-1 at several sites [[44], [45], [46]]. HS has the ability to promote multimerization of fibrillin molecules prior to furin cleavage of newly secreted fibrillin [44,47] and fibrillin multimers also bind HS more strongly [48]. At higher concentrations, addition of heparin/HS is known to inhibit microfibril deposition [45,46,49]. HS is available through post-translational modifications on both membrane bound receptors, such as syndecans and glypicans, and non-membrane bound ECM molecules such as perlecan and agrin, where the interaction with perlecan and fibrillin-1 is via both HS and non-HS perlecan domains [50].

### Requirement for fibronectin for microfibril assembly

Interactions with HS containing syndecans in conjunction with integrin binding of the RGD containing TB4 domain of fibrillin both influence fibrillin assembly. The fibrillin-1 RGD region primarily interacts with integrin α5β1 on fibroblasts but interactions with integrin αvβ3 were seen on other cell types [12], and it has been found that only fibrillin-1 fragments containing the RGD sequence support cell adhesion, for a variety different cell types including mesenchymal stem cells, chondrocyte progenitors and induced pluripotent stem cells [51,52]. However, unlike fibronectin, fibrillin does not need integrin mediated cell adhesion to assemble if fibronectin is also present [42]. Fibrillin microfibrils precede the evolution of the RGD site by 500 million years [53], indicating that the RGD site is not needed for microfibril assembly in invertebrates. The RGD site, integrins and fibronectin are only seen in vertebrate biology, and mutations affecting the TB4 domain of fibrillin result in altered microfibril deposition causing stiff skin syndrome [54], which can be recapitulated in mice by mutating the RGD site to RGE [55], suggesting for vertebrate microfibril deposition other more complex factors are needed via integrin mediated cell signalling. Indeed, it has also been found that interactions with the RGD site of fibrillin-1 by fibroblasts, control the expression of over one hundred microRNAs, some of which regulate TGFβ signalling (including miR-503) and focal adhesion formation (including miR-612 and miR-3185) [56]. Ancestral Fibrillin also preceded elastin which first appeared in gnathostomes (jawed vertebrates), so fibrillin did not evolve to assemble elastin. However the evolution of elastin which also led to the appearance of closed circulatory systems [57,58], also coincided with the divergence of ancestral fibrillin to fibrillin-1 and fibrillin 2/3 [53], although this period of duplication also known as 2R resulted in a large number of genetic changes.

**Fig. 3:**
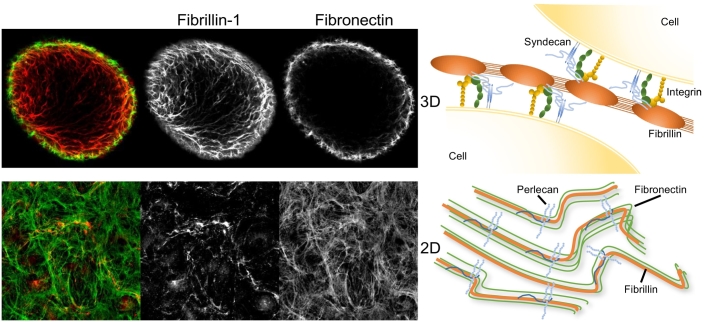
Fibrillin deposition in 3D and 2D cell cultures. Human dermal fibroblasts were cultured in either non-adherent U bottom wells (top) or on glass coverslips (bottom) for 6 days. Fibrillin deposition (red) is seen throughout the whole spheroid, while fibronectin (green) is primarily on the outer cell layers. In comparison, fibrillin is entirely colocalised with fibronectin in cells in 2D culture. Also shown, in the right handside panel, are schematic diagrams illustrating fibrillin interactions with cell surface receptors, such as integrins and HS containing proteoglycans such as surface bound syndecans and ECM bound perlecan, in 3D (top) and 2D cultures (bottom).

In mesenchymal cells such as fibroblasts, fibrillin is deposited on the fibronectin network, and knockdown of fibronectin results in perturbation of fibrillin deposition in 2D cell cultures [59,60]. However In 3D mesenchymal cell cultures, fibronectin is located almost entirely in the outer cell layers, whilst fibrillin both colocalises with fibronectin on the outer cell layers but is also abundantly deposited in the tightly cell packed inner volume [61]. This suggests that fibrillin deposition can switch between fibronectin directed or cell surface interaction directed depending on the extracellular environment (Fig. 3).

Close cellular contact is also seen in epithelial cells sheets, where fibronectin is not required but does enhance deposition. In retinal pigmented epithelial cells, syndecan 4 as well as integrins α5β1 or α8β1 are required for microfibril deposition, and fibronectin is not needed but does enhance deposition [62]. The same study showed inhibition of cadherin junctions also disrupted microfibril deposition in retinal pigmented epithelial cells, and was independent of fibronectin deposition, indicating that the close cell-cell contact is needed for this mode of fibrillin deposition. It has been proposed that HS-rich cell junctions promote microfibril assembly, where fibronectin is not available [63].

### Role of ADAMTS and ADAMTSL proteins in microfibril assembly

Members of the A Disintegrin And Metalloprotease with Thrombospondin type-1 repeats (ADAMTS) and ADAMTS-Like (ADAMTSL) family have been implicated in fibrillin microfibril assembly which influences elastic fibre formation. ADAMTS10 co-localises with microfibrils and enhances their deposition in vitro [64]. It is required for focal adhesion formation via interactions with both heparan sulphate and fibronectin [65]. Contrary to this, ADAMTS6 depletes HS reducing the number of focal adhesion complexes, required for fibrillin-1 microfibril deposition [65]. Therefore, the modulatory effects of both ADAMTS6 and -10 on focal adhesions play a vital role in microfibril deposition and subsequent elastic fibre assembly. Mouse models with disruption to ADAMTS10 show an accumulation of fibrillin-2 microfibrils in various tissues suggesting fibrillin-2 could be a substrate of ADAMTS10 [66,67]. ADAMTS17 co-localises with fibrillin-1, -2 and fibronectin fibres, binds to the N- and C-termini of fibrillin-2 but does not cleave either fibrillin-1 or -2 [68].

ADAMTS-Like proteins do not have catalytic activity but their interactions and localisation with fibrillin microfibrils support a regulatory role in microfibril formation and deposition. ADAMTSL2 binds to fibrillin-1 and the N-terminal binding site encompasses a fibrillin-1 mutation which results in Weill Marchesani Syndrome (WMS) [69]. ADAMTSL2 also binds to fibrillin-2 and deletion of ADAMTSL2 results in an accumulation of fibrillin-2 microfibrils [70]. ADAMTSL4 forms an independent fibrillar network which co-localises with fibrillin-1 microfibrils and enhances microfibril biogenesis [71,72]. Although interaction studies have demonstrated binding of ADAMTSL5 to fibrillin-1 and fibrillin-2, as well as its co-localisation with microfibrils, there is no evidence of ADAMTSL5 having a direct role in microfibril assembly [73]. On the other hand, ADAMTSL6α and -β variants promote fibrillin-1 microfibril biogenesis and localise with fibrillin-1 microfibrils in elastic and non-elastic tissues [74].

### Tissue assembly of fibrillin microfibrils

In tissues, fibrillin microfibrils are organised into larger tissue specific structures which are important for the mechanical properties of that tissue. In the reticular dermis of skin, fibrillin-1 is formed into thick horizontally arranged elastic fibres which are connected to the dermal-epidermal junction through perpendicularly arranged bundles of microfibrils called elaunin and oxytalan fibres [75]. In normal skin, fibrillin-2 is only detected at the dermal-epidermal-junction and in vessel walls but has increased expression in wound healing [76]. In the lung, elastic fibres form branched networks which surround the alveoli allowing for elastic recoil during breathing and in the medial layer of the aorta, elastic fibres form sheets which surround layers of smooth muscle cells. In the ciliary zonule of the eye, microfibrils are found in the absence of elastin, here they bundle together to form ciliary zonule fibres. These fibres then form larger bundles which span between the ciliary body and the lens and deform the lens during accommodation. The large bundles of ciliary zonule fibres are held together by perpendicularly arranged ciliary zonule fibres which wrap around their circumference [29,77]. Microfibrils in the ciliary zonule are connected via bridging complexes [29,33], two candidates proteins for this role are ADAMTSL4 [78] and LTBP2 [79] which when absent result in ciliary zonule disruption.

## Role of fibrillin in elastogenesis

### Developmental expression of fibrillin and tropoelastin

In elastic tissues, elastic fibre assembly commences during early gestation with the expression and deposition of fibrillin microfibrils. In humans, fibrillin-1, -2 and -3 are expressed during development [80]; however, fibrillin-1 expression is the most abundant and dominates from late morphogenesis to adult life [81]. Moreover, proteomic data show that fibrillin isoform expression is both species and tissue dependent [82,83]. Fibrillin-3 is predominantly expressed in developing tissues, but the fibrillin-3 gene is disrupted in mice, so may be less important in mammals [8]. In developing elastic and non-elastic tissues, fibrillin-1 and -2 co-express and co-assemble in the matrix [84] and the requirement for both fibrillin-1 and fibrillin-2 isoforms has been demonstrated by a double knockout mouse model with early embryonic lethality [85]. Fibrillin microfibril deposition in developing elastic tissues precedes the expression of the elastin precursor, tropoelastin. Tropoelastin is highly expressed during embryogenesis, in comparison to the low levels detected in adult tissues [[86], [87], [88]].

### The role of fibrillin in elastic fibre assembly

The assembly of elastic fibres in the ECM is a highly organised and multifaceted process requiring the expression and contribution of several ECM proteins.

The amorphous core of insoluble elastic fibres is comprised of cross-linked tropoelastin, glycosaminoglycans such as HS [89] and proteoglycans such a biglycan [90] deposited on a scaffold of co-assembled fibrillin-1 and -2 microfibrils [3]. Tropoelastin secretion is aided by elastin binding protein, an inactive splice variant of β-galactosidase [91], where C-terminal interactions with cell surface heparan and chondroitin sulphates mediate the coacervation of tropoelastin into larger dense globules [92,93]. Interactions between the hydrophobic regions of individual monomers results in the alignment of lysine residues that are enzymatically cross-linked by lysyl oxidase (LOX) and LOX-like 1 (LOXL1) to form larger aggregates [[94], [95], [96]]. Recently, we have shown that LOXL2 interacts with tropoelastin and LOXL2 catalyses the deamination of tropoelastin resulting in cross-linked tropoelastin peptides. The elastin-like material generated by LOXL2 was resistant to trypsin proteolysis and displayed mechanical properties similar to mature elastin. As LOXL2 co-distributes with elastin in the vascular wall this suggests that LOXL2 could participate in elastogenesis in vivo [97].

Once cross-linked, tropoelastin aggregates interact with co-assembled fibrillin-1 and -2 microfibrils near the cell surface via defined interactive domains. Tropoelastin interacts with microfibril associated glycoprotein-1 (MAGP1) [98,99] and MAGP1 interacts with the N-terminal region of fibrillin-1, suggesting tethering of tropoelastin to microfibrils [98,100]. A similar role for microfibrillar-associated protein 4 (MFAP4) is likely as it also binds to fibrillin and tropoelastin and promotes tropoelastin assembly [101]. Tropoelastin also directly interacts with the N-terminal region of fibrillin-1 via the TB2 domain and a covalent cross-link between tropoelastin and fibrillin is formed by transglutaminase-2 (TG2) [100,102]. The cross-link between the fibrillin TB2 domain and tropoelastin was mapped to residues Q669 in fibrillin and K38 in domain 4 of tropoelastin and addition of this region of fibrillin enhanced the coacervation of tropoelastin [103]. There is also a second high affinity binding site in the central region of fibrillin-1 encompassing the TB3 domain [102]. The initial deposition of tropoelastin aggregates onto fibrillin microfibrils is believed to be the primary step required for further coacervation and an augmentation of tropoelastin globule recruitment to form larger elastic fibres [103]. Once deposited on to fibrillin microfibrils, the tropoelastin aggregates coalesce to form stable, insoluble elastic fibres.

## Microfibril binding proteins that facilitate elastogenesis

### LTBPs in elastic fibre assembly

**Fig. 4:**
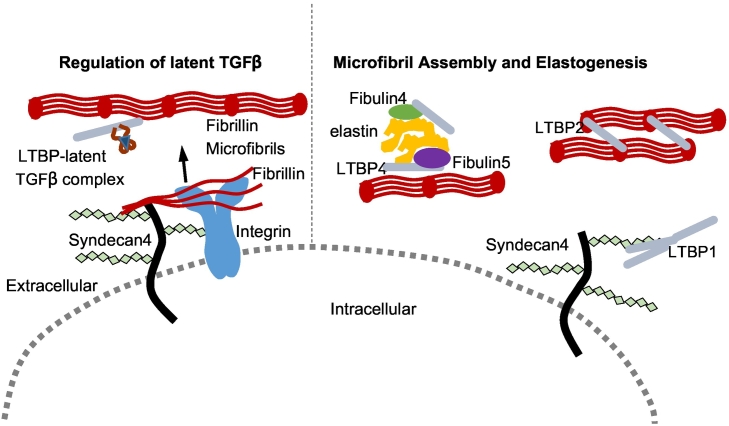
Schematic diagram illustrating the dual roles of the LTBP family in TGFβ regulation and matrix assembly. Shown on the left, LTBP1, -3 and -4 form a large latent TGFβ complex which binds fibrillin to sequester TGFβ in the matrix. In TGFβ-independent mechanisms, shown on the right, LTBP1 can multimerize which is enhanced by HS (potentially on syndecans) and stabilised by TG2 cross-linking, LTBP2 stabilises fibrillin bundles and LTBP4 binds fibulin-4 and -5 to aid in deposition of elastin aggregates on fibrillin microfibrils in elastogenesis.

The LTBPs are extracellular glycoproteins in the fibrillin superfamily with similar modular domain structure [[104], [105], [106], [107]]. There are four LTBPs with LTBP1, -3 and -4 having important roles in the processing and secretion of TGFβ [108]. LTBP2 does not interact with TGFβ [109], but has a role in stabilising fibrillin microfibril bundles in the eye [79]. Indeed, dual functionalities have been proposed for members of the LTBP family, in either secretion of TGFβ or microfibril assembly and elastogenesis [110] (Fig. 4). LTBP1 has both long and short isoforms generated by alternate splicing at the N-terminus [111,112], as does LTBP4 [107,113]. Both LTBP2 and -4 have been implicated in the formation of elastic fibres in elastogenesis and LTBP2 and -4 have some commonalities in their functional roles, as LTBP4 can compensate for LTBP2 in some tissues [114]. However, even though LTBP3 and -4 are both expressed in the lung, they can only partially compensate for each other in lung development suggesting that the LTBP family have only some over-lapping functions [115].

Fibrillin is required for the deposition of LTBP2, -3 and -4 [116] but LTBP1 can be assembled in the absence of fibrillin [116]. In contrast, LTBP1 requires fibronectin for its deposition [116,117] and interacts with fibronectin [118]. Binding of LTBP1 to fibulin-4 may act as an additional mediator for fibrillin microfibril association [49,119]. The C-terminal region of LTBP1, -2 and -4 binds to fibrillin [120,121] but an equivalent region of LTBP3 does not interact with fibrillin [120] despite fibrillin being required for its deposition. LTBP1, -2 and -4 bind HS [[122], [123], [124]], suggesting they may interaction with HS proteoglycans (HSPGs) such as syndecans.

LTBP1 is a substrate for transglutaminase type II (TG2) and can be cross-linked into the matrix to enhance latency of the latent TGFβ complex [125,126]. Our recent study showed that both the N- and C-terminus of LTBP1 are substrates for TG2 and intermolecular N—N and N—C cross-links can form [122]. Oligomerisation of LTBP1 can occur in the absence of other proteins and is calcium dependent. LTBP1 multimerization is promoted by HS and stabilised by TG2 crosslinking suggesting a mechanism whereby LTBP1 filaments could support the bridging of fibrillin microfibrils to the matrix.

LTBP2 colocalises with elastic fibres and the deposition of LTBP2 into the matrix is dependent on the presence of a preformed fibrillin-1 network as fibrillin-1 knockdown disrupts LTBP2 deposition [127,128]. LTBP2 has been suggested to regulate elastic fibre formation in a fibrillin-independent manner via fibulin-5, and can compete for the fibulin-5 tropoelastin interaction potentially displacing elastin assemblies in elastogenesis [129,130]. LTBP3 is expressed in heart, skeletal muscle, prostate and ovaries [131] with interactions with fibrillin-1 important for matrix incorporation [116], although colocalisation of LTBP3 has been shown with fibronectin [118]. Secretion of LTBP3 requires co-expression with TGFβ and is dependent on binding to LAP [131,132].

Several studies have investigated the elastogenic role of LTBP4 where a dual functionality for LTBP4 has been proposed as a regulator of either elastic fibre assembly or TGFβ levels in lung [110,133]. LTBP4 is important in elastic fibre assembly with LTBP4 mutations resulting in Urban-Rifkin-Davis Syndrome (URDS) also known as autosomal recessive cutis laxa type 1C [134] which is replicated in knockout mouse models [135,136]. A lack of LTBP4 results in abnormal distribution and formation of elastic fibres [134,136]. Fibroblasts from URDS patients show increased TGFβ activity [134], consistent with data from *LTBP4*^−/−^ mice [135,137]. However, reducing the level of TGFβ does not normalize elastogenesis indicating that LTBP4 has a dual functionality in regulating both elastogenesis and TGFβ signalling and the role of LTBP4 in elastogenesis is TGFβ independent [110].

### Fibulin-4 and fibulin-5 in elastogenesis

Several elastic-fibre associated proteins are involved in the different stages of elastogenesis, from the micro-assembly of tropoelastin coacervates to the macro-assembly of elastic fibres. The fibulins are a multifunctional family of ECM glycoproteins comprising both long (fibulin-1, -2, -6) and short fibulins (fibulin-3, -4, -5, -7) [138]. Fibulin-1, -2, -3, -4, and -5 bind tropoelastin [139] and fibulin-2, -3, -4, and -5 are involved in elastic fibre formation. In particular, fibulin-4 and fibulin-5 (also known as developing arteries and neural crest EGF-like (DANCE)) play an important role in elastogenesis. Fibulin-4 and fibulin-5 null mice lack well-developed elastic fibres, demonstrating the importance of both glycoproteins in elastic fibre formation [[140], [141], [142]]. Functional studies have shown that fibulin-4 binds LOX with high affinity, which further results in a stronger interaction with tropoelastin, suggesting a role for fibulin-4 in the recruitment of LOX and the coacervation of tropoelastin [143,144]. Fibulin-5 has a strong interaction with tropoelastin and interacts with LOX, LOXL1, LOXL2 and LOXL4 to induce elastic fibre assembly and cross-linking [96,143,145]. Additionally, both fibulin-4 and -5 bind to the N-terminal region of fibrillin-1, suggesting a role in chaperoning tropoelastin aggregates to fibrillin microfibrils and facilitating the formation of cross-linked elastic fibres [139,143].

LTBP4 colocalises to and interacts with fibulin-5 via the four-cysteine domain at its N-terminus which binds to the C-terminal region of fibulin-5 [133]. LTBP4-fibulin interactions are important for elastogenesis as LTBP4 knockdown disrupts fibulin-5 and tropoelastin deposition which can be rescued by addition of LTBP4 [133]. These findings indicate that LTBP4 is essential for proper deposition of fibulin-5-elastin complexes onto fibrillin microfibril scaffolds. LTBP4 also interacts with fibulin-4 and fibulin-4 deposition is disrupted in LTBP4^−/−^ mice but normal in LTBP4S^−/−^ mice that express the LTBP4L isoform [136,146]. This raised the question whether the different LTBP4 isoforms differentially bind fibulin-4. Indeed mice expressing only the LTBP4L isoform with reduced expression of fibulin-4 die during the early postnatal period [146]. In contrast LTBP4S^−/−^;fibulin-4^R/R^ mice survive to adulthood [135,147], indicating that LTBP4L interacts with fibulin-4 and this interaction is essential for survival [146]. Together these findings imply that the elastogenic role of LTBP4 is both fibulin-4 and fibulin-5 dependent and that fibulin-5 cannot compensate for fibulin-4. Moreover, LTBP4S favours binding to fibulin-5 [133], whereas LTBP4L favours binding to fibulin-4 [136]. Overall, both LTBP4 isoforms are required for deposition of both fibulin-5-elastin and fibulin-4-elastin complexes onto fibrillin microfibrils.

### The Emilins in elastic fibre formation

Emilin (Elastin Microfibril Interface-Located ProteIN)-1 and -2 are found at sites where elastin and fibrillin microfibrils are in close proximity [148]. The Emilins are deposited on and co-regulated with fibrillin-1 [149] and they are required for elastic fibre formation. They have a regulatory role in cell adhesion, migration and wound healing, and have been implicated in blood vascular morphology, tumour cell invasiveness and dermal proliferation [[149], [150], [151]]. Fibulin-4 has been identified as an Emilin-1 binding partner with Emilin-1 knockdown preventing fibulin-4 deposition, suggesting a requirement for Emilin-1 in fibulin-4 incorporation in the matrix [152].

## Final considerations

Fibrillin is large, modular, extracellular matrix glycoprotein that assembles into beaded microfibrils which have roles in elastic fibre assembly, elastic tissue function and extracellular signalling events. In most tissues, fibrillin microfibrils associate with elastin to form elastic fibres and hence make key contributions to the elastic function of these tissues acting as a stiff reinforcer of elastin-containing tissues. Fibrillin microfibrils also provide limited elasticity in tissues devoid of elastin. They also provide a multifunctional platform for the interaction of many matrix molecules required for elastic fibre assembly and function and provide a connection to the cell surface. Fibrillin is needed for the correct assembly of many microfibril associated proteins, including members of the LTBP family, and mediate interactions between fibulin-4 or fibulin-5 and LTBPs or LOX/L enzymes facilitating their functions. Indeed fibrillin microfibril and elastic fibre biology is highly complex which provides a challenge to the research community to unravel the multiple molecular and cellular interactions that underpin elastogenesis. However, understanding these events provides a future opportunity to inform future regenerative medicine strategies and intervene in disease processes.
